# The Spectroscopy Study of the Binding of an Active Ingredient of *Dioscorea* Species with Bovine Serum Albumin with or without Co^2+^ or Zn^2+^

**DOI:** 10.1155/2014/247595

**Published:** 2014-06-04

**Authors:** He-Dong Bian, Xia-Lian Peng, Fu-Ping Huang, Di Yao, Qing Yu, Hong Liang

**Affiliations:** ^1^Key Laboratory of Development and Application of Forest Chemicals of Guangxi, Guangxi University of Nationalities, Nanning 530006, China; ^2^Key Laboratory for the Chemistry and Molecular Engineering of Medicinal Resources (Ministry of Education of China), School of Chemistry and Pharmaceutical Sciences of Guangxi Normal University, Guilin 541004, China

## Abstract

Diosgenin (DIO) is the active ingredient of *Dioscorea* species. The interaction of DIO with bovine serum albumin (BSA) was investigated through spectroscopic methods under simulated physiological conditions. The fluorescence quenching data revealed that the binding of DIO to BSA without or with Co^2+^ or Zn^2+^ was a static quenching process. The presence of Co^2+^ or Zn^2+^ both increased the static quenching constants *K*_SV_ and the binding affinity for the BSA-DIO system. In the sight of the competitive experiment and the negative values of Δ*H* ^0^ and Δ*S* ^0^, DIO bound to site I of BSA mainly through the hydrogen bond and Van der Waals' force. In addition, the conformational changes of BSA were studied by Raman spectra, which revealed that the secondary structure of BSA and microenvironment of the aromatic residues were changed by DIO. The Raman spectra analysis indicated that the changes of conformations, disulfide bridges, and the microenvironment of Tyr, Trp residues of BSA induced by DIO with Co^2+^ or Zn^2+^ were different from that without Co^2+^ or Zn^2+^.

## 1. Introduction

**Figure 1:**
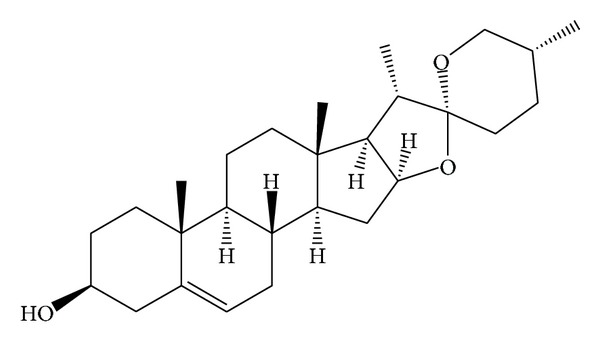
The chemical structure of diosgenin.

Dioscoreaceae mainly distributes Guangdong, Sichuan, and Zhejiang provinces in China. Diosgenin (DIO), 3**β**-hydroxy-5-spirostene (Figure 1), one of the active ingredients of* Dioscorea *species, is derived from the tubers of* Dioscorea* species. The previous studies have indicated that DIO can retard the progression of osteoporosis [1] and attenuate plasma cholesterol [2], possess anti-inflammatory [3] and inhibition of vasoconstriction [4] effects, and so on.

Serum albumin (SA) is composed of three structurally homologous domains (I–III); each domain contains two subdomains (A and B). It is the major transport protein, which can act as a carrier of endogenous and exogenous ligands [5]. The transportation and distribution of the drugs in vivo are related to their interaction with serum albumin. On the other hand, the binding of drugs also can change the conformation function of serum albumin. So it is important to investigate the interaction between drugs and serum albumin. Bovine serum albumin (BSA) has similar structure and property with human serum albumin (HSA); the major difference between these two serum albumins was that there was only one Trp residue in HSA, but in BSA there were two Trp residues (^134^Trp and ^212^Trp). Compared with HSA, BSA was always selected as a model protein due to its low cost, unusual ligand-binding properties. The study of the interaction between drugs and BSA plays an important role in pharmacology and pharmacodynamics [6].

Blood plasma contains many metal ions which play important roles in the biochemical processes. The previous reports of interactions between serum albumin and several metal ions suggested that many metal ions have special binding sites on proteins [7–9]. The binding of drugs with serum albumin in the presence of metal ions was also extensively studied [10, 11]. The previous studies indicated that the presence of metal ions would not only affect the interaction of serum albumin with drugs, but also the conformational changes of serum albumin induced by drugs. The metal ions of Co^2+^ and Zn^2+^ are abundant essential elements in organism which possess many biochemical functions. It is necessary to investigate the interaction of BSA-DIO in the presence of Co^2+^ or Zn^2+^.

In this paper, we studied the binding of DIO with BSA under simulated physiological conditions of pH = 7.43. Fluorescence spectra and Raman spectra were employed to investigate the binding process and the changes of protein structure in the absence and presence of Co^2+^ or Zn^2+^.

## 2. Materials and Methods

### 2.1. Materials

DIO (98%, purchased from Aladdin Reagent Company) was dissolved in ethanol to prepare a stock solution of 1 × 10^−3^ mol·L^−1^. 0.05 mol·L^−1^ phosphate buffer solution (PBS) of pH = 7.43, contained 0.1 mol·L^−1^ NaCl. BSA (98%, fatty acid free, and globulin free, Sigma) was dissolved in PBS to prepare stock solution of 1 × 10^−3^ mol·L^−1^ and stored at 277 K, diluted before used. Ibuprofen and ketoprofen were dissolved in ethanol to prepare stock solution of 1 × 10^−3^ mol·L^−1^, respectively. Sodium chloride, ethanol, zinc chloride, cobalt(II) chloride hexahydrate, and other experimental drugs are analytically pure reagents. Double distilled water was used throughout.

### 2.2. Fluorescence Spectrum

Fluorescence spectra were carried out on a RF-5301 fluorescence spectrophotometer (Japan Shimadzu Company). A solution of 5.0 × 10^−7^ mol·L^−1^ BSA was added in a 1.0 cm quartz cell; metal ions or DIO were then gradually added into BSA by microinjector. Scan the solution of BSA in the absence and presence of DIO or metal ions in the wavelength range of 300–500 nm, respectively. The slit widths were 5 nm/5 nm; the excitation wavelength was 280 nm. The reaction temperatures for DIO-BSA system without metal ions were controlled at 291 K, 298 K, and 306 K, respectively. The reaction temperatures for DIO-BSA system with Co^2+^ or Zn^2+^ were controlled at 298 K. For site marker experiment, BSA and site markers were mixed in equimolar concentrations at room temperature for 2 h, and then DIO was gradually added into the solution, scan the fluorescence spectra of the solution.

#### 2.2.1. The Quenching Mechanism

In order to confirm the quenching mechanism induced by DIO, the fluorescence quenching was described by Stern-Volmer equation [12]:

(1) $$\begin{array}{c} \frac{F_{0}}{F}=1+k_{q}\tau_{0}\left[Q\right]=1+K_{\text{SV}}\left[Q\right], \end{array}$$

where *F*_0_ and *F* are the fluorescence intensity in the absence and presence of quencher, respectively. *k*_*q*_ is the quenching rate constant. *τ*_0_ is the average fluorescence lifetime of the biomolecule in the absence of quencher. [*Q*] is the concentration of quencher. *K*_SV_ is the Stern-Volmer quenching constant. Since the fluorescence lifetime of the biopolymer is 10^−8^ s [13], *K*_SV_ and *k*_*q*_ can be obtained according to the slopes of the Stern-Volmer plots.

#### 2.2.2. The Quenching Mechanism and the Binding Constant

The binding constants of the static quenching were calculated according to the modified Stern-Volmer equation [14]:

(2) $$\begin{array}{c} \frac{F_{0}}{\left(F_{0}-F\right)}=\frac{1}{f}+\frac{1}{\left(Kf\left[Q\right]\right)}, \end{array}$$

where *f* is the fraction of accessible fluorescence and *K* is the effective quenching constant for the accessible fluorophores, which are analogous to associative binding constants.

#### 2.2.3. Thermodynamic Parameters

The enthalpy change (Δ*H* ^0^) was regarded as a constant when the temperature changed little, then enthalpy change (Δ*H* ^0^) and entropy change (Δ*S* ^0^) can be obtained from Van't Hoff equation [15]:

(3) $$\begin{array}{c} \ln K=-\frac{\Delta H^{0}}{RT}+\frac{\Delta S^{0}}{R}, \end{array}$$

(4) $$\begin{array}{c} \Delta G^{0}=\Delta H^{0}-T\Delta S^{0}=-RT\ln K, \end{array}$$

where *R* was the gas constant and Δ*G* ^0^ was the standard free energy change.

#### 2.2.4. Energy Transfer Calculation

According to Forster's nonradiative energy transfer theory [16, 17], the energy transfer efficiency is decided not only by the distance between the acceptor and donor, but also the critical energy transfer distance (*R*_0_); that is [18],

(5) $$\begin{array}{c} E=1-\frac{F}{F_{0}}=\frac{R_{0}^{6}}{R_{0}^{6}+r^{6}}, \end{array}$$

where *r* is the distance between acceptor and donor and *R*_0_ is the critical distance in the case of the transfer efficiency is 50%

(6) $$\begin{array}{c} R_{0}^{6}=8.8\times 10^{-25}K^{2}n^{-4}\Phi J, \end{array}$$

where *K* ^2^ is the spatial orientation factor of the dipole, *n* is the refractive index of the medium, *Ф* is the fluorescence quantum yield of donor, and *J* is the overlap integral of fluorescence emission spectrum of donor and absorption spectrum of acceptor

(7) $$\begin{array}{c} J=\frac{\sum F\left(\lambda \right)\varepsilon \left(\lambda \right)\lambda^{4}\Delta \lambda }{\sum F\left(\lambda \right)\Delta \lambda }, \end{array}$$

where *F*(*λ*) is the fluorescence intensity of the donor at wavelength *λ* and *ε*(*λ*) is the molar absorption coefficient of the acceptor at wavelength *λ*.

### 2.3. Raman Spectrum

The Raman spectra were recorded on a Renishaw Invia+Plus FT-Raman spectrometer using an Ar^+^ laser with excitation wavelength of 514 nm. The laser power was 3 mW; the recording range was 200–2000 cm^−1^ with spectral resolution of 1 cm^−1^. Scan the Raman spectra of 5 × 10^−4^ mol·L^−1^ BSA in the absence and presence of DIO and metal ions of the same concentration under the room temperature. In Raman experiment, DIO was first dissolved in ethanol/water (1 : 9) and then mixed with BSA, metal ions solution to prepare Raman scanning sample. The curve fitting of Raman spectral regions was analysed by the curve-fitting procedure (Peak Analyzer module of Origin 8.0, Microcal Origin, USA) using Gaussian curves.

## 3. Results and Discussion

### 3.1. The Influence of DIO on the Fluorescence of BSA without or with Co^2+^ or Zn^2+^

**Figure 2:**
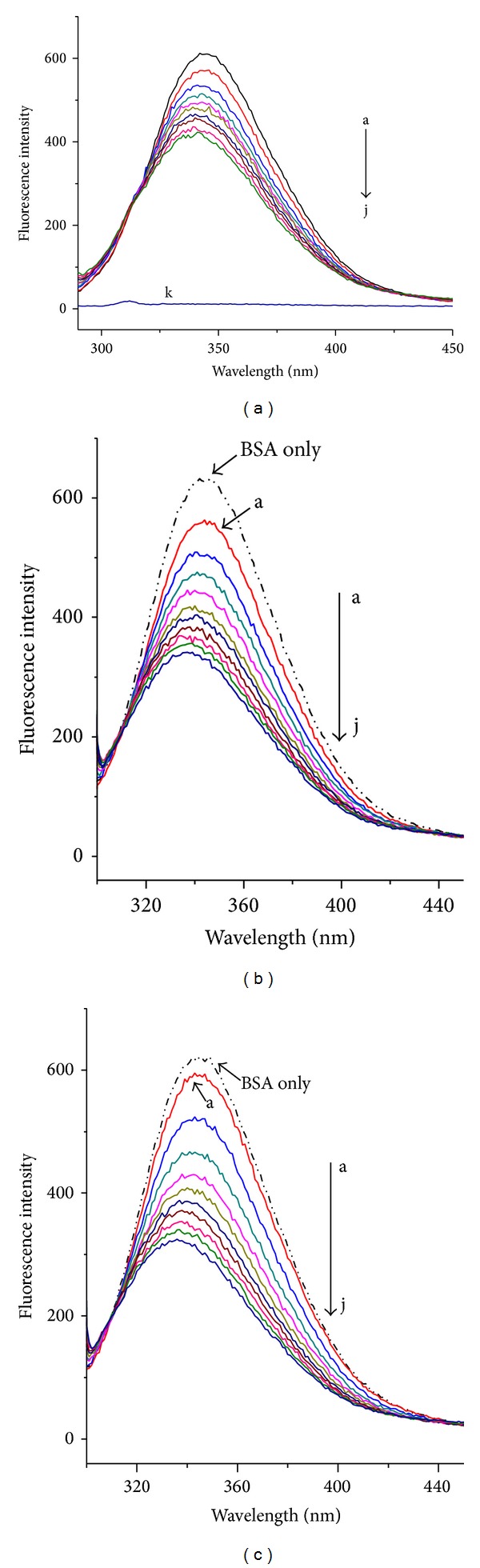
The fluorescence spectra of BSA by DIO in the absence and presence of Co^2+^ or Zn^2+^. (a) BSA-DIO system; (b) BSA-DIO-Co^2+^ system; (c) BSA-DIO-Zn^2+^ system. From a to j, the concentration of DIO was varied from 0 to 9 × 10^−6^ mol·L^−1^ at increments of 1 × 10^−6^ mol·L^−1^. k: DIO only, 9 × 10^−6^ mol·L^−1^. [BSA] = [Co^2+^] = [Zn^2+^] = 5 × 10^−7^ mol·L^−1^, *T* = 298 K.

For macromolecules, the fluorescence measurements can give information of the binding of small molecule substances to protein. When excited at 280 nm, the intrinsic fluorescence of BSA was mainly contributed by Trp residues [19, 20]. The fluorescence of BSA quenched by DIO in the presence and absence of Co^2+^ or Zn^2+^ of the same concentration was shown in Figure 2.  Figure 2(a) showed that the fluorescence intensity of BSA decreased regularly with increasing DIO. Meanwhile, the small blue shift observed with increasing DIO concentration indicated a more hydrophobic environment of the fluorescence chromophore of BSA [21]. Figures 2(b) and 2(c) showed that the fluorescence intensity decreased after adding Co^2+^ or Zn^2+^ with the same concentration. These indicated that the metal ions bind with BSA which is in accordance with our previous work. When DIO was added into BSA solution containing equimolar Co^2+^ or Zn^2+^, the fluorescence intensity decreased regularly with blue shift. The shapes of spectra were similar to those in the absence of Co^2+^ or Zn^2+^, while the fluorescence intensity in the presence of Co^2+^ or Zn^2+^ was weaker than those without Co^2+^ or Zn^2+^. The result obtained suggested that the fluorescence was quenched not only by the metal ions but also by DIO. The interaction occurred among BSA, DIO, and the metal ions.

### 3.2. The Quenching Mechanism and the Binding Constant

Fluorescence quenching is classified as dynamic quenching and static quenching. Usually, static quenching is due to the formation of ground-state complex between fluorophore and quencher. The static quenching constant will decrease with increasing temperature, because higher temperature will lower the stability of the complex. Dynamic quenching results from collision between fluorophore and quencher, as higher temperatures result in larger diffusion coefficients, so the reverse effect is observed [22–24].

**Figure 3:**
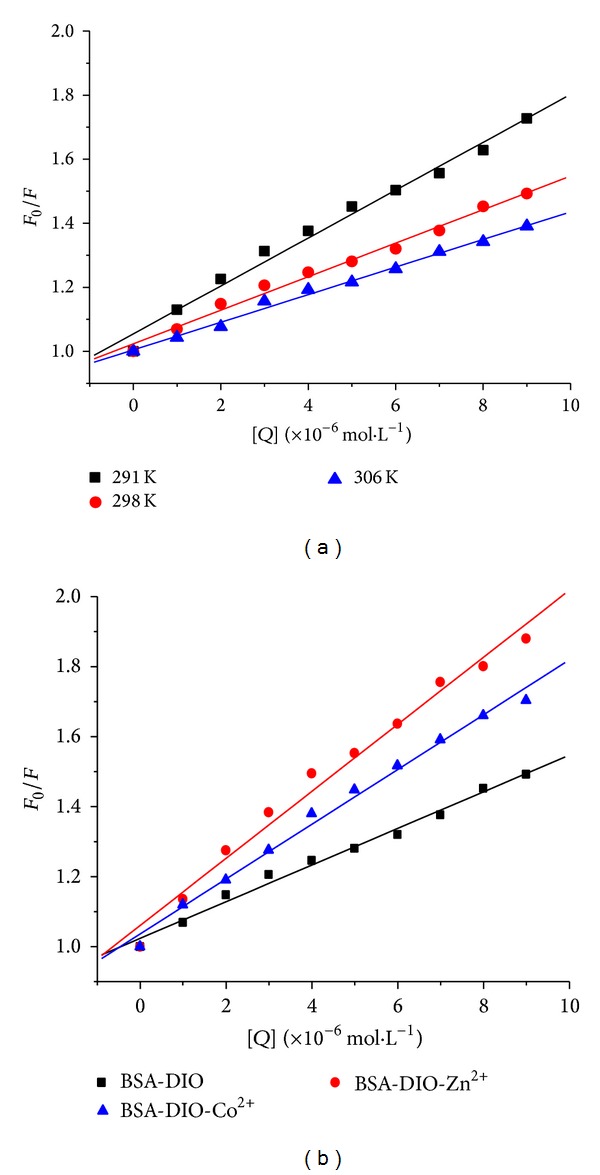
The Stern-Volmer plots for the binding of DIO with BSA at different temperatures (a) and in the absence and presence of Co^2+^ or Zn^2+^, *T* = 298 K (b).

To confirm the quenching mechanism, the fluorescence quenching data were analyzed according to the Stern-Volmer equation (1). The Stern-Volmer plots of different temperatures and the corresponding results were shown in Figure 3 and Table 1. The results showed that *K*_SV_ decreased with increasing temperature, and *k*_*q*_ were much greater than 2.0 × 10^10^ L·mol^−1^·s^−1^, indicating a static quenching mechanism between BSA and DIO [25]. The quenching constants *K*_SV_ were both increased in the presence of Co^2+^ or Zn^2+^, indicating that the presence of Co^2+^ or Zn^2+^ increased the fluorescence quenching effect of DIO. Meanwhile, the *k*_*q*_ values in the presence of Co^2+^ or Zn^2+^ suggest a static quenching mechanism for the binding of DIO to BSA with Co^2+^ or Zn^2+^.

**Figure 4:**
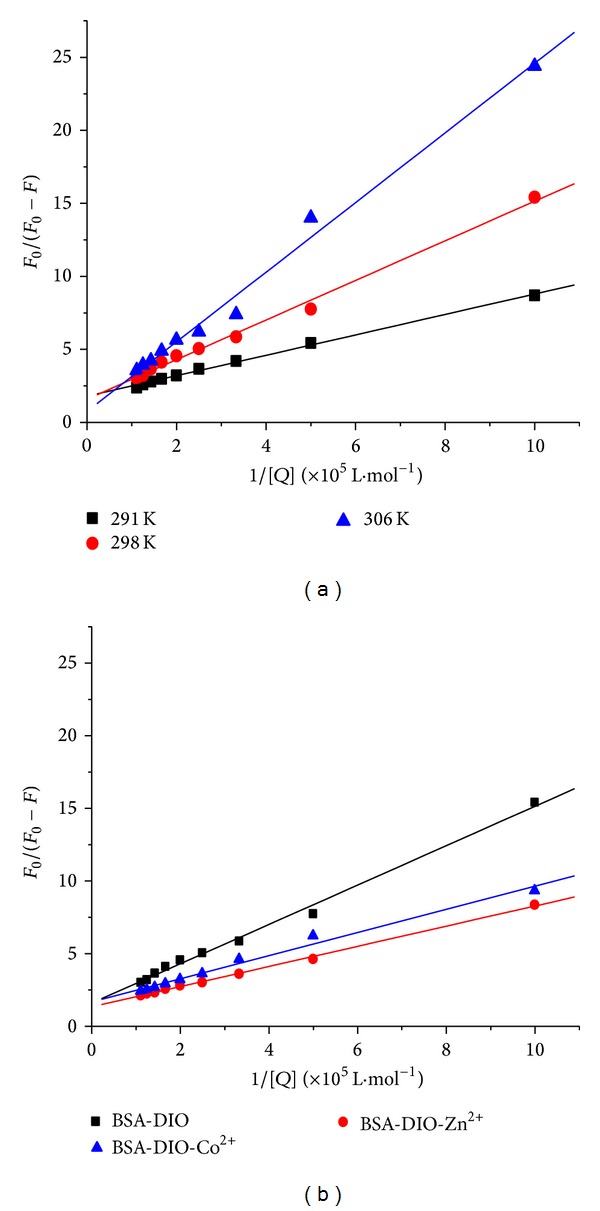
The modified Stern-Volmer plots for the binding of DIO with BSA at different temperatures (a) and in the absence and presence of Co^2+^ or Zn^2+^, *T* = 298 K (b).

In order to obtain the binding constants, the experimental data were also analyzed according to the modified Stern-Volmer equation (2).  Figure 4 showed the modified Stern-Volmer plots at different temperatures, and the calculated binding constants *K* for BSA-DIO system were listed in Table 2. The *K* values for the binding of DIO with BSA were decreased with increasing temperature, which further suggested that the binding of DIO with BSA was static quenching. The binding constants *K* for BSA-DIO system in the presence of Co^2+^ or Zn^2+^ were calculated to be 2.10 × 10^5^ L·mol^−1^ and 1.94 × 10^5^ L·mol^−1^, respectively. The binding constants *K* for BSA-DIO system were both increased in the presence of Co^2+^ or Zn^2+^, implying stronger binding of DIO to BSA in the present of Co^2+^ or Zn^2+^.

### 3.3. The Nature of the Binding Forces

**Figure 5:**
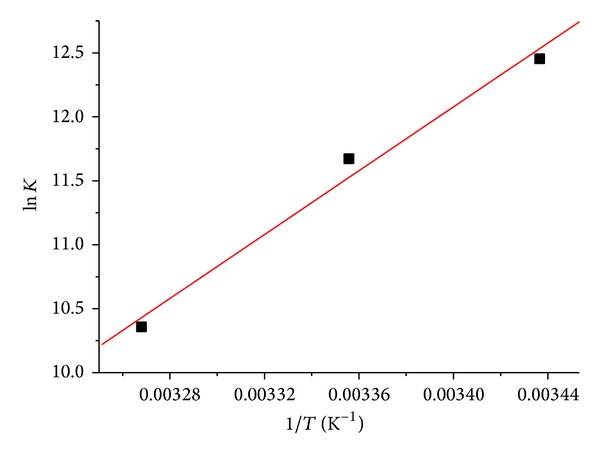
Van't Hoff plots for the binding of DIO with BSA.

Generally, small organic molecules bound to biomolecules mainly through four types of acting forces: hydrogen bond, van der Waals' force, electrostatic force, and hydrophobic interaction, and so forth [26]. The force type can be determined by three thermodynamic parameters, enthalpy (Δ*H* ^0^), free-energy change (Δ*G* ^0^), and the entropy change (Δ*S* ^0^). These parameters for the interaction between DIO and BSA were calculated by Van't Hoff equation (3) and thermodynamic equation (4). The Van't Hoff plots were shown in Figure 5, and the thermodynamic parameters were listed in Table 2. The negative Δ*G* ^0^ suggested that the reactions between DIO and BSA were spontaneous. DIO bound to BSA mainly through the hydrogen bond and Van der Waals' force as evidenced by the negative value of Δ*H* ^0^ and Δ*S* ^0^ [27].

### 3.4. Energy Transfer between Drugs and BSA

**Figure 6:**
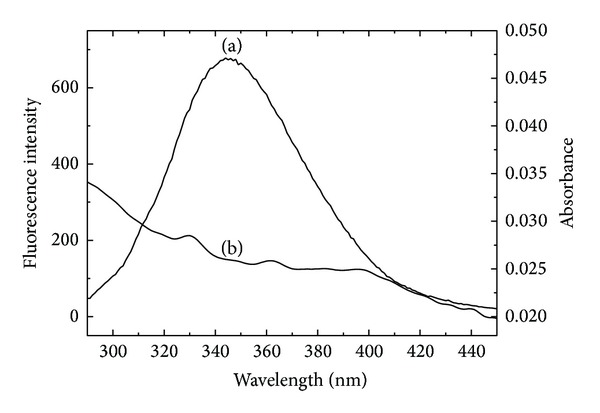
Spectral overlap between the fluorescence emission spectrum of BSA and the absorption spectrum of DIO. (a) fluorescence emission spectrum of BSA (5.0 × 10^−7^ mol·L^−1^); (b) absorption spectrum of DIO (5.0 × 10^−7^ mol·L^−1^).

The overlap of the absorption spectrum of DIO and the fluorescence emission spectrum of BSA is shown in Figure 6. For BSA, *K* ^2^ = 2/3, Φ = 0.15, and *n* = 1.336 [28]; then we can obtain the following results: *J* = 8.36 × 10^−14^ cm^3^·L·mol^−1^, *R*_0_ = 3.64 nm, and *r* = 5.36 nm. The distance between BSA and DIO was smaller than 8 nm, which suggested that the quenching of BSA by DIO was static quenching, which was in accordance with the results above.

### 3.5. The Binding of Site Maker Probes

There were two major binding sites for drugs on albumin which were known as Sudlow sites I and II. Site I is formed as a pocket in subdomain IIA and involves the lone tryptophan of the protein (^212^Trp). Site I is adaptable and binds kinds of ligands with very different chemical structures. Site II locate at subdomain IIIA. It can bind smaller ligands because it is smaller, less flexible, and narrower than site I [29, 30]. Site I showed affinity for warfarin, ketoprofen, and so forth, and site II for ibuprofen, flufenamic acid, and so forth [31–33]. In order to determine the binding sites of DIO to BSA, the competitive displacement experiments were carried out using different site probes of ketoprofen for site I and ibuprofen for site II, respectively. The results showed that the binding constants of DIO to BSA were surprisingly changed from 2.57 to 1.304 × 10^5^ L·mol^−1^ in the presence of ketoprofen, while the *K* values almost remained the same in the case of ibuprofen (2.47 × 10^5^ L·mol^−1^). The results indicated that ketoprofen exhibited significant displacement of DIO. However, ibuprofen was not displaced by DIO obviously. These meant that the binding site of DIO to BSA was site I.

### 3.6. Analysis of BSA Conformational Changes

**Figure 7:**
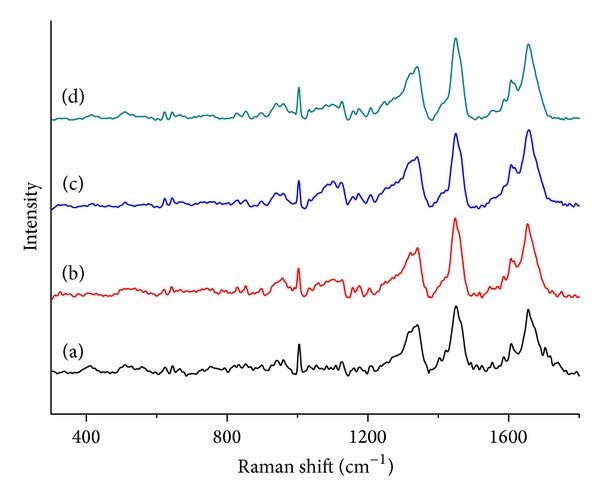
Raman spectra of (a) free BSA; (b) DIO-BSA system; (c) DIO-BSA-Co^2+^ system; (d) DIO-BSA-Zn^2+^ system. [BSA] = [DIO] = [Co^2+^] = [Zn^2+^] = 5 × 10^−4^ mol·L^−1^.

**Figure 8:**
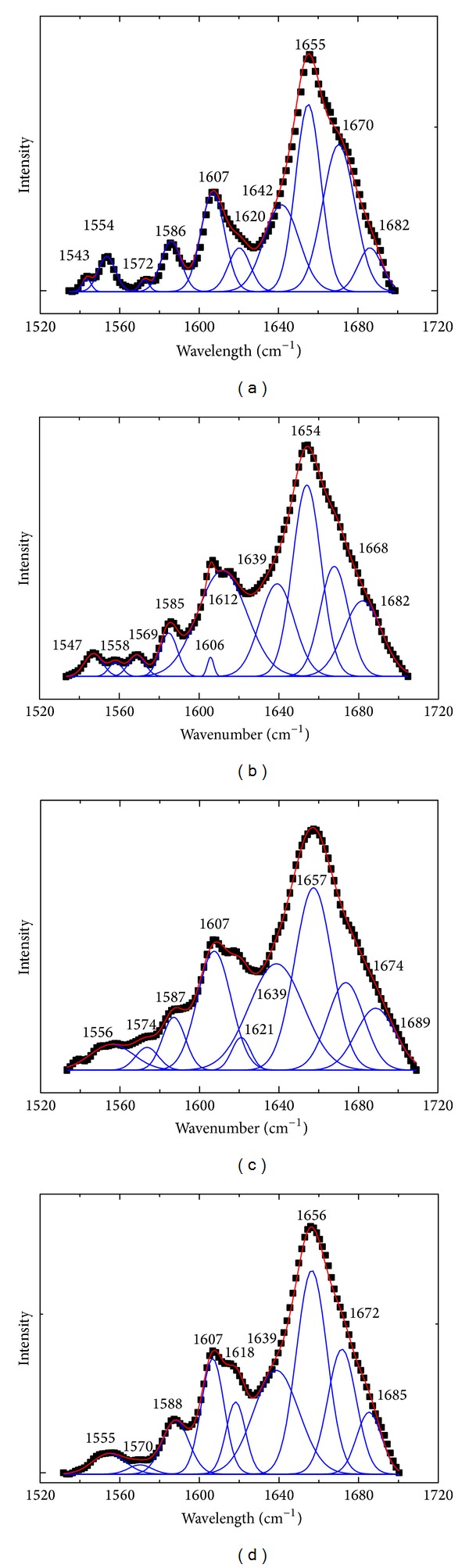
The curve fitting of Raman amide I of (a) free BSA; (b) DIO-BSA system; (c) DIO-BSA-Co^2+^ system; (d) DIO-BSA-Zn^2+^ system. The experimental spectra (black dots); the fitting curves (solid line).

Raman spectroscopy has emerged as a useful method to investigate the conformational changes of protein secondary structure and the microenvironment of amino acid residues [34]. In order to study the effects of DIO on the conformation of BSA, we analyzed the amide I and the regions of aromatic amino acid residues of Raman spectra. In Raman spectra, the peaks that appeared in the region of 1550–1620 cm^−1^ were the ring vibration bands of aromatic residues [35]. The amide I band (1700–1630 cm^−1^) originated mainly from peptide C=O stretching [36, 37].  Figure 7 displayed the Raman spectra of BSA and BSA-DIO system in the absence and presence of Co^2+^ and Zn^2+^. In the amide I band of BSA, the major band of BSA around 1648–1658 cm^−1^ was the characterization of *α*-helix; while the band of 1630–1640 cm^−1^ represented short segment chains connecting the *α*-helix, the bands of *β*-turn were centered at 1680–1700 cm^−1^, respectively [38–42].  Figure 8 was the curve fitting of Raman amide I; the corresponding results were listed in Table 3. The results showed that native BSA contains major *α*-helix conformation (55.71%), which are consistent with the previous ones reported for BSA by Raman, infrared, and CD spectroscopy [43–45]. The *α*-helix contents decreased to 47.58% due to the binding of DIO. Meanwhile, the content of *β*-turn increased while the content of short segment decreased. The results indicated that the secondary structure of BSA was changed by DIO. Competing with the BSA-DIO system, the decreased extent of *α*-helix content was lower in the presence of Zn^2+^, while for Co^2+^ there was an increase. The results indicated that the presence of Co^2+^ or Zn^2+^ affects the changes of BSA secondary structure.

**Figure 9:**
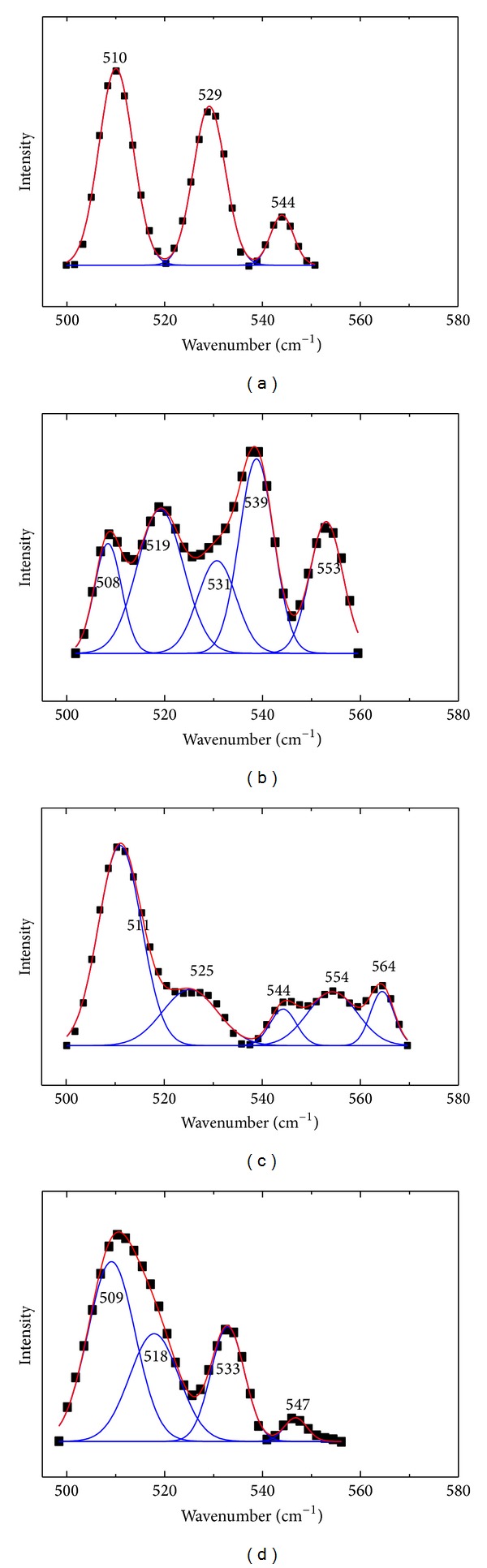
The analysis of the S-S bands of (a) free BSA; (b) DIO-BSA system; (c) DIO-BSA-Co^2+^ system; (d) DIO-BSA-Zn^2+^ system. The experimental spectra (black dots), the fitting curves (solid line).

The conformation of 17 disulphide bridges of serum albumin molecule can be sensitively determined by Raman spectroscopy. The disulphide bridges of BSA have three conformations: gauche-gauche-gauche (g-g-g, peaks around 510 cm^−1^), gauche-gauche-trans or trans-gauche-gauche (g-g-t or t-g-g, peaks around 525 cm^−1^), and trans-gauche-trans (t-g-t, peaks around 540 cm^−1^) [46].  Figure 9 was the analysis of the S-S bands; the conformations of 17 disulphide bridges were obtained according to the fitting results. As shown in Table 4, the major conformation of disulphide bridges in native BSA was g-g-g conformations. After binding DIO, there were 7 S-S bridges converted conformations, while 3 conformations of S-S bridges were changed in the presence of Co^2+^ or Zn^2+^, which indicated the presence of Co^2+^ or Zn^2+^ decreased the changed of DIO to S-S bridges.

**Figure 10:**
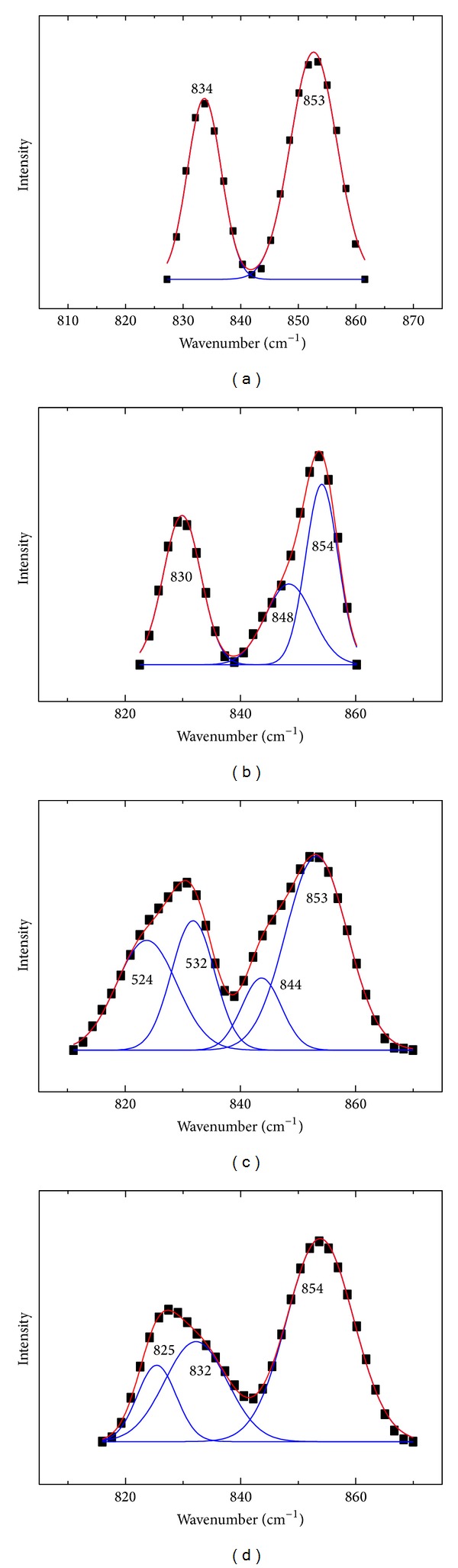
The analysis of the Tyr side chains of (a) free BSA; (b) DIO-BSA system; (c) DIO-BSA-Co^2+^ system; (d) DIO-BSA-Zn^2+^ system. The experimental spectra (black dots), the fitting curves (solid line).

The tyrosyl doublet around 850 and 830 cm^−1^, so-called “Fermi-resonance Tyr-doublet, was due to the symmetric ring-breathing vibration and the out-of-plane ring-bending vibration. The bands at 850 and 830 cm^−1^ are extremely sensitive to hydrogen bonding of the phenolic OH-groups, and the intensity ratio of this doublet (*I*_850_/*I*_830_) is an indicator of the microenvironment of tyrosine residues. The value of this ratio between 0.3 and 0.5 indicated that the tyrosy1 residues were “buried.” On the other hand, the tyrosine residues were “exposed,” when the values range from 1.25 to 1.40 [47, 48]. The analysis of the Tyr side chains was displayed in Figure 10; the results in Table 5 showed that the value of *I*_850_/*I*_830_ decreased after the addition of DIO. But *I*_850_/*I*_830_ for BSA-DIO-Co^2+^ and BSA-DIO-Zn^2+^ systems were both increased in the presence of Co^2+^ or Zn^2+^, and the values were larger than that of free BSA. The results indicated that the buriedness of Tyr residues in protein was increased by DIO, but the presence of Co^2+^ or Zn^2+^ decreased the buriedness of Tyr residues [49].

**Figure 11:**
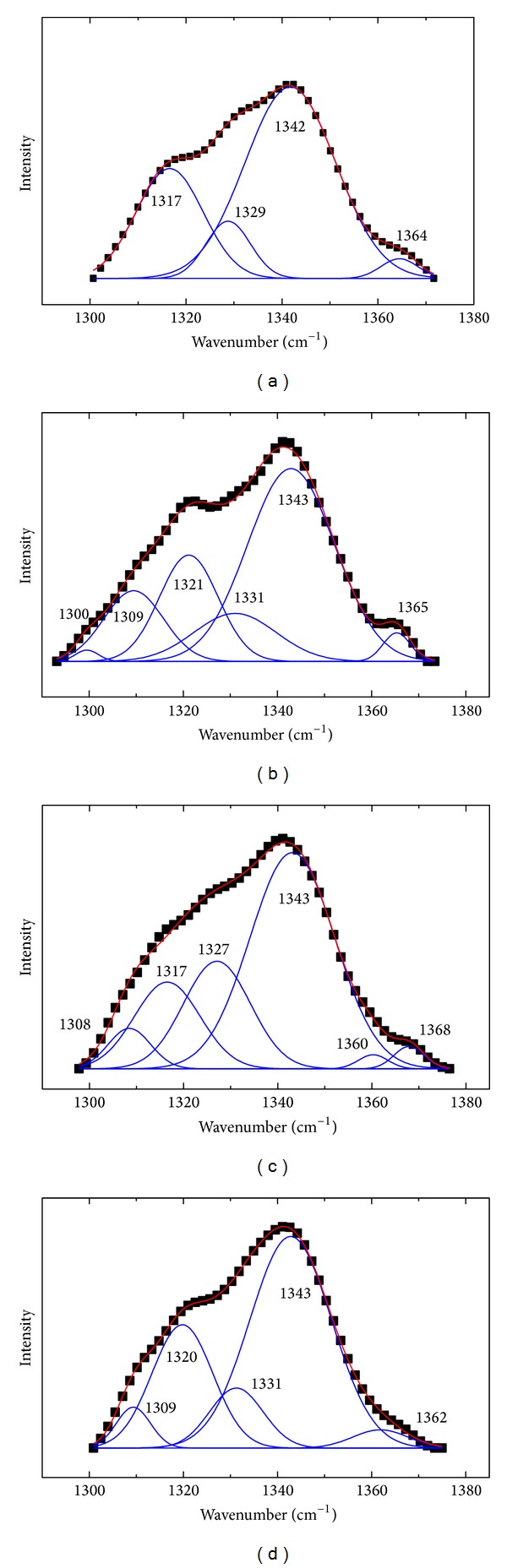
The analysis of the Trp side chains of (a) free BSA; (b) DIO-BSA system; (c) DIO-BSA-Co^2+^ system; (d) DIO-BSA-Zn^2+^ system. The experimental spectra (black dots), the fitting curves (solid line).

The band appeared around 1340 cm^−1^ and the weak shoulder around 1363 cm^−1^ owing to the Fermi-resonance doublet bands of Trp residues. Their intensity ratio *I*_1363_/*I*_1340_ can also be used to investigate the overall hydrophobicity of the environment surrounding tryptophan residues [50, 51].  Figure 11 was the analysis of the Trp side chains; the results were listed in Table 5. The intensity ratio of *I*_1363_/*I*_1340_ increased from 0.0409 to 0.0461 due to the addition of DIO, while greater changes were found in BSA-DIO-Co^2+^ and BSA-DIO-Zn^2+^ systems. The results indicated that the hydrophobicity around the Trp residues increased due to the binding of DIO; the hydrophobicity increased more in the presence of Co^2+^ and Zn^2+^ [51].

## 4. Conclusions

In summary, we simulated the interaction of DIO with BSA in vitro by spectroscopic investigations. The experimental results indicated that the drugs could bind with BSA to form a DIO-BSA complex. The binding reaction was spontaneous. DIO bound to site I of BSA mainly through the hydrogen bond and Van der Waals' force. The presence of Co^2+^ or Zn^2+^ increased the quenching effect and the binding affinity of DIO to BSA. Otherwise, the analysis of conformation change confirmed that the binding of DIO induced the unfolding of protein secondary structure. Although the changes of BSA secondary structure caused by DIO in the presence of Co^2+^ or Zn^2+^ were different from those without metal ions, they all major led to the decrease of *α*-helix conformation. The addition of DIO changed 7 conformations of S-S bridges of BSA, while the changes were both reduced to 3 in the presence of Co^2+^ or Zn^2+^. Besides, DIO increased the buriedness of Tyr residues in protein, but the effects were opposite for BSA-DIO-Co^2+^ and BSA-DIO-Zn^2+^ systems. Meanwhile, the hydrophobicity around the tryptophan residues was all increased due to the binding of DIO in the absence and presence of Co^2+^ or Zn^2+^.

## Figures and Tables

**Table 1:** The Stern-Volmer quenching constants of DIO with BSA.

	*T* (K)	*K*_SV_ (×10^4^·mol^−1^)	*k*_*q*_ (×10^12^ L·mol^−1^·s^−1^)	*R* ^a^	S.D.^b^
DIO-BSA	291	7.47	7.47	0.9928	0.03
	298	5.23	5.23	0.9947	0.02
	306	4.31	5.23	0.9964	0.01

BSA-DIO-Co^2+^	298	7.83	4.31	0.9958	0.02

BSA-DIO-Zn^2+^	298	9.58	7.83	0.9923	0.04

^a^ *R* is the correlation coefficient; ^b^S.D. is the standard deviation for the *K*_SV_ values.

**Table 2:** The static binding constants *K* and thermodynamic parameters of DIO with BSA at different temperatures.

	*T* (K)	*K* (×10^5^ L·mol^−1^)	*R* ^a^	Δ*G* ^0^ (kJ·mol^−1^)	Δ*S* ^0^ (J·mol^−1^·K^−1^)	Δ*H* ^0^ (kJ·mol^−1^)
DIO-BSA	291	2.57	0.9985	−30.31	−252.48	−103.78
	298	1.17	0.9971	−28.54		
	306	0.32	0.9948	−26.52		

^a^ *R* is the correlation coefficient for the *K* values.

**Table 3:** The curve fitting results of Raman amide *I* of BSA.

System		*α*-Helix	Short segment	*β*-Turn
BSA	Frequency (cm^−1^)	1655	1642	1682
	Content (%)	55.71	34.51	9.78

BSA-DIO	Frequency (cm^−1^)	1654	1639	1682
	Content (%)	47.58	27.70	24.72

BSA-DIO-Co^2+^	Frequency (cm^−1^)	1657	1639	1689
	Content (%)	45.55	38.52	15.93

BSA-DIO-Zn^2+^	Frequency (cm^−1^)	1657	1639	1685
	Content (%)	51.48	38.30	10.22

**Table 4:** The conformation of the 17 disulfide bridges of BSA.

System	g-g-g	g-g-t or t-g-g	t-g-t	change
BSA	9	7	1	—
BSA-DIO	2	9	6	7
BSA-DIO-Co^2+^	12	4	1	3
BSA-DIO-Zn^2+^	8	5	4	3

**Table 5:** The analysis of the Tyr and Trp side chains.

System	BSA	BSA-DIO	BSA-DIO-Co^2+^	BSA-DIO-Zn^2+^
*I*_850_/*I*_830_	1.7180	1.0352	2.1477	2.1645
*I*_1363_/*I*_1340_	0.0409	0.0461	0.0502	0.0574
